# A Putative Role for Ubiquitin-Proteasome Signaling in Estrogenic Memory Regulation

**DOI:** 10.3389/fnbeh.2021.807215

**Published:** 2022-01-25

**Authors:** Sarah B. Beamish, Karyn M. Frick

**Affiliations:** Department of Psychology, University of Wisconsin-Milwaukee, Milwaukee, WI, United States

**Keywords:** 17β-estradiol, memory, hippocampus, protein degradation, proteasome, ubiquitin

## Abstract

Sex steroid hormones such as 17β-estradiol (E_2_) are critical neuromodulators of hippocampal synaptic plasticity and hippocampus-dependent memory in both males and females. However, the mechanisms through which E_2_ regulates memory formation in both sexes remain unclear. Research to date suggests that E_2_ regulates hippocampus-dependent memory by activating numerous cell-signaling cascades to promote the synthesis of proteins that support structural changes at hippocampal synapses. However, this work has largely overlooked the equally important contributions of protein degradation mediated by the ubiquitin proteasome system (UPS) in remodeling the synapse. Despite being critically implicated in synaptic plasticity and successful formation of long-term memories, it remains unclear whether protein degradation mediated by the UPS is necessary for E_2_ to exert its beneficial effects on hippocampal plasticity and memory formation. The present article provides an overview of the receptor and signaling mechanisms so far identified as critical for regulating hippocampal E_2_ and UPS function in males and females, with a particular emphasis on the ways in which these mechanisms overlap to support structural integrity and protein composition of hippocampal synapses. We argue that the high degree of correspondence between E_2_ and UPS activity warrants additional study to examine the contributions of ubiquitin-mediated protein degradation in regulating the effects of sex steroid hormones on cognition.

## Introduction

The sex steroid hormone 17β-estradiol (E_2_) is the most potent and prevalent circulating estrogen and has been studied extensively in the field of hormones and cognition because of its ability to regulate hippocampal synaptic plasticity, spinogenesis, and the storage of long-term memories in males and females. In the early 1990’s, seminal work demonstrated that dendritic spine density on pyramidal neurons in the CA1 region of the dorsal hippocampus (DH) fluctuates throughout the rat estrous cycle (Woolley et al., 1990), suggesting for the first time that endogenous sex steroid hormones, such as E_2_, alter structural plasticity in brain regions relevant for cognition. Research since then has demonstrated that exogenous E_2_ can increase CA1 dendritic spine density in ovariectomized rats and mice as quickly as 30 min following systemic injection (MacLusky et al., 2005; Inagaki et al., 2012) or DH infusion (Tuscher et al., 2016). Likewise, exogenous E_2_ also increases intrinsic excitability, excitatory neurotransmission, and long-term potentiation (LTP) in hippocampal neurons (Wong and Moss, 1992; Woolley et al., 1997; Foy et al., 1999; Foy, 2001). These, among other E_2_-induced enhancements in hippocampal synaptic function and spinogenesis, are thought to underlie E_2_’s ability to facilitate the consolidation of multiple hippocampus-dependent memories, including spatial, object recognition, fear, and social memories in both males and females (Tuscher et al., 2015; Taxier et al., 2020).

Despite the ample evidence that E_2_ enhances hippocampal function and memory formation in both sexes, the neural mechanisms through which E_2_ exerts its effects remain poorly understood. A growing body of research has examined how E_2_ activates rapid cell-signaling events to drive increases in protein synthesis to support structural changes at hippocampal synapses (Sarkar et al., 2010; Fortress et al., 2013; Sellers et al., 2015; Tuscher et al., 2016). These studies suggest that local protein synthesis is necessary for E_2_ to both increase CA1 spine density and enhance memory consolidation (Fortress et al., 2013; Tuscher et al., 2016). However, this work has largely overlooked the potentially vital and parallel contribution of protein degradation mediated by the ubiquitin proteasome system (UPS). In the UPS, proteins are tagged with ubiquitin and become substrates for degradation by the 26S proteasome. UPS-mediated protein degradation is a necessary counterpart to protein synthesis in driving synaptic plasticity and memory (Jarome and Helmstetter, 2013; Hegde, 2017) because it regulates the destruction of proteins that impose inhibitory constraints on synaptic remodeling, cell signaling, and gene transcription events across subcellular compartments of the neuron (Hegde, 2004).

In this review, we discuss the view that UPS-mediated protein degradation is an overlooked mechanism that may play a key role in regulating E_2_’s beneficial effects on hippocampal plasticity and memory. The review briefly summarizes E_2_’s effects on hippocampal function and then describes in some detail how the UPS functions to influence memory. Effects of E_2_ on UPS activity are then discussed, as are the numerous ways in which E_2_ and UPS signaling overlap to potentially regulate hippocampal function, which include regulation of the structural integrity and protein composition of hippocampal synapses. Finally, we offer some suggestions for future research.

## Estradiol and Hippocampal Function

**Figure 1:**
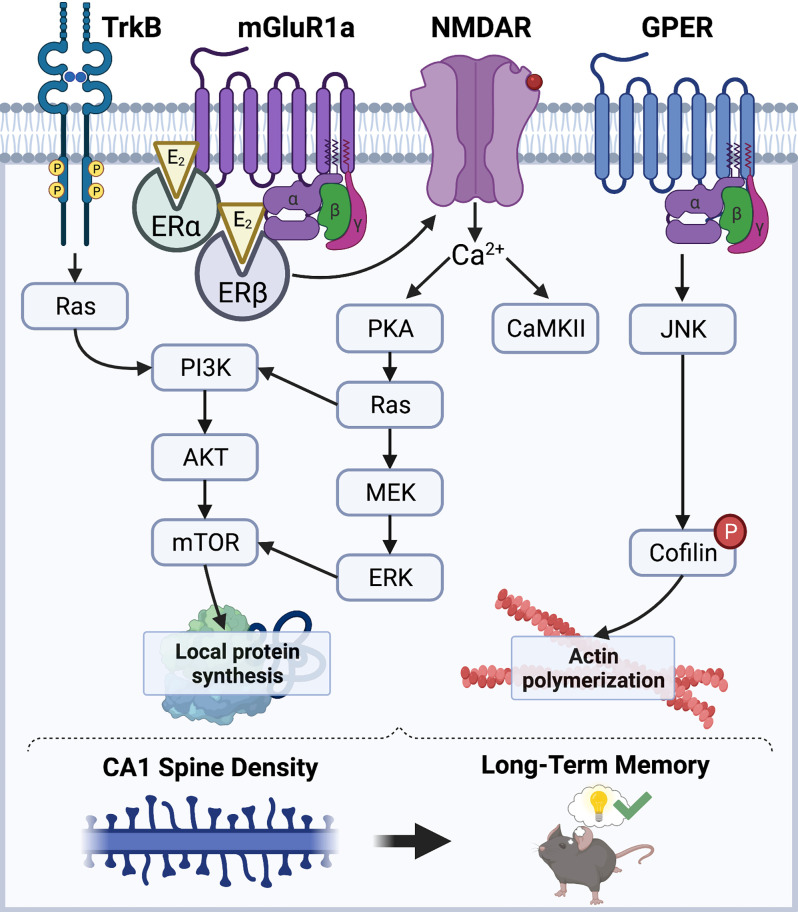
Schematic diagram illustrating a model of the mechanisms through which E_2_ regulates CA1 spine density and memory. E_2_ acts *via* membrane-associated receptors like mGluRs, GPER, and TrkB, as well as ion channels like NMDAR, to stimulate cell-signaling kinases that promote local protein synthesis and actin polymerization. ERα and ERβ promote protein synthesis *via* ERK and mTOR signaling, whereas GPER promotes actin polymerization through JNK signaling. Illustration created using BioRender.com.

E_2_ has received considerable attention in the past three decades for its role as a powerful modulator of hippocampal synaptic morphology, plasticity, and long-term memory in males and females of various mammalian species (Frick, 2015; Hojo et al., 2015; Hamson et al., 2016; Taxier et al., 2020). However, the mechanisms through which E_2_ promotes hippocampal synaptic function in both sexes remain largely unclear. Work to date has demonstrated that E_2_ facilitates hippocampal synaptic plasticity and memory consolidation in ovariectomized female rodents by acting at the plasma membrane, where it interacts with membrane-bound estrogen receptors (ERs; Boulware et al., 2005, 2013) to initiate signal transduction events to rapidly modulate synaptic morphology (Figure 1). Estrogen receptors alpha and beta (ERα and ERβ) are the canonical intracellular estrogen receptors. Although ERα and ERβ are known for exerting genomic effects in the nucleus, they are also abundantly expressed throughout all segments of hippocampal neurons, including axon terminals, dendrites, and dendritic spines (Milner et al., 2000, 2005), where they are positioned near the plasma membrane to interact with metabotropic glutamate receptors (mGluRs) and other receptors to rapidly regulate synaptic signaling (Mitterling et al., 2010). Data from our lab and others indicate that activation of ERα or ERβ facilitates the consolidation of object recognition and spatial memories in ovariectomized rats and mice (Jacome et al., 2010; Kim and Frick, 2017; Hanson et al., 2018; Fleischer et al., 2021). Additional evidence suggests that glutamate receptors play key roles in mediating these memory-enhancing effects, as ERα and ERβ directly interact with mGluR1a to trigger extracellular signal-regulated kinase (ERK) signaling in the DH to facilitate object placement and object recognition memory consolidation in ovariectomized mice (Boulware et al., 2013). E_2_ may also interact with the membrane-bound G-protein-coupled estrogen receptor (GPER) to enhance spatial and object recognition memories, however, GPER agonism appears to facilitate consolidation independently by phosphorylating c-Jun N-terminal kinase (JNK), not ERK (Kim et al., 2016), suggesting that GPER and E_2_ use different cell-signaling pathways to regulate memory formation. Additional signaling mechanisms that regulate E_2_’s memory-enhancing and spinogenic effects depend on the rapid activation of NMDA receptors (NMDARs) and tyrosine receptor kinase B (TrkB) to trigger downstream signaling cascades including calcium/calmodulin-dependent protein kinase II (CaMKII), protein kinase A (PKA), ERK, and phosphoinositide 3-kinase (PI3K; Murakami et al., 2006; Fernandez et al., 2008; Lewis et al., 2008; Fan et al., 2010; Gross et al., 2021).

E_2_-induced enhancements in hippocampal synaptic plasticity and memory formation have largely been attributed to its rapid effects on CA1 dendritic spine density (Mukai et al., 2007; Inagaki et al., 2012; Tuscher et al., 2016; Kim et al., 2019). E_2_ can promote hippocampal LTP by regulating actin polymerization (Kramár et al., 2009), which is necessary for spine growth and maturation (Penzes and Cahill, 2012). In the DH of ovariectomized mice, E_2_ rapidly and transiently increases phosphorylation of cofilin (Kim et al., 2019), which leads to actin stabilization and polymerization (Chen et al., 2007; Babayan and Kramár, 2013). E_2_-induced increases in CA1 spine density also require the rapid synthesis of new proteins within the postsynaptic density (PSD). Several studies have demonstrated that E_2_ can activate ERK and Akt signaling to promote local protein synthesis by activating mechanistic target of rapamycin (mTOR) and mTOR complex 1 (mTORC1) signaling (Akama and McEwen, 2003; Sarkar et al., 2010; Fortress et al., 2013; Briz and Baudry, 2014). Interestingly, E_2_ increases local protein synthesis of the synaptic scaffolding molecule PSD-95 in cultured neurons in an ERα-, Akt-, and mTOR-dependent manner (Akama and McEwen, 2003), suggesting that these newly synthesized proteins contribute directly to the expanding dendritic architecture. Furthermore, work from our lab suggests that E_2_ acts at membrane-localized ERs in the DH to activate ERK and mTORC1 signaling, which is necessary for E_2_ to increase CA1 spine density and to enhance object recognition and object placement memory consolidation in ovariectomized female mice (Boulware et al., 2013; Fortress et al., 2013; Tuscher et al., 2016), thereby supporting the hypothesis that local protein synthesis is necessary for E_2_-induced spinogenesis and memory.

Thus, evidence to date suggests that E_2_ promotes hippocampus-dependent memory by inducing rapid membrane-initiated cell-signaling events that increase CA1 dendritic spine density by reorganizing components of the cytoskeleton, as well as driving increases local protein synthesis. These findings reflect the field’s historic focus on identifying the signaling mechanisms that promote protein production to support structural changes at hippocampal synapses. However, this attention on protein synthesis has caused researchers to overlook the potential contributions of protein degradation as an equal, but opposite, regulator of E_2_’s effects on memory. As will be discussed below, protein degradation mediated by the UPS plays a vital role in hippocampal plasticity and memory by structurally remodeling the synapse and degrading proteins that exert inhibitory constraints to synaptic plasticity. We show that the signaling mechanisms that facilitate proteasomal protein degradation overlap considerably with those that regulate E_2_’s effects on CA1 spine density and memory, suggesting compelling reasons to explore protein degradation mechanisms as key mediators of E_2_’s effects on memory.

## The Ubiquitin-Proteasome System

**Figure 2:**
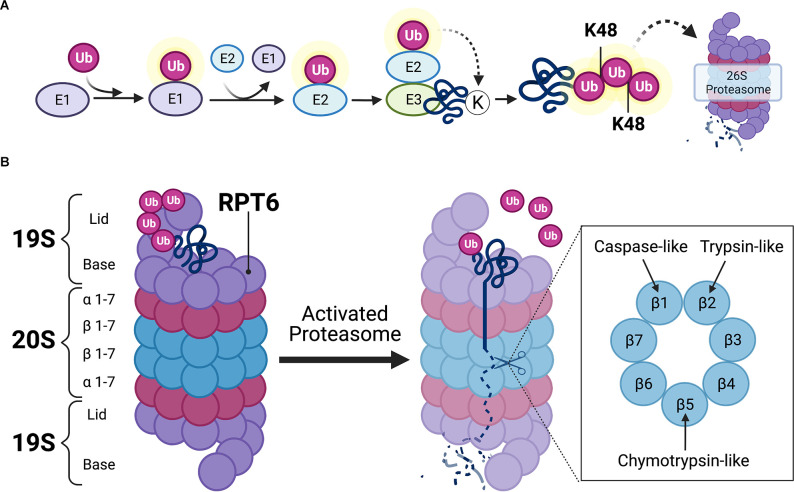
Ubiquitin-mediated proteolysis. **(A)** Schematic illustration of ubiquitin targeting pathway. Free ubiquitin molecules are activated by E1-activating ligases *via* ATP hydrolysis. Activated ubiquitin is then bound to E1, which then transfers the active ubiquitin molecule to E2 carrier enzymes. E3 ligases bind to target substrates destined for degradation. E2 enzymes localize to the E3-substrate complex, enabling the transfer of active ubiquitin to substrate protein. Additional ubiquitin molecules attach at lysine-48 (K48) residues on subsequent ubiquitin, eventually forming a K48-linked polyubiquitin chain that is destined to the 26S proteasome for proteasomal degradation. **(B)** Detailed schematic view of the 26S proteasome. The 26S proteasome contains one or two 19S caps and a 20S core. K48-linked polyubiquitinated protein binds to the 19S regulatory cap at ubiquitin binding sites. Upon activation of the proteasome, which usually occurs at the 19S subunit Rpt6 (Ser^120^ residue), the polyubiquitin chain becomes hydrolyzed and the substrate protein unfolds to allow β subunits in the 20S core to hydrolyze proteins *via* caspase-, trypsin-, and chymotrypsin-like activities. Illustration created using BioRender.com.

The UPS is the primary mechanism for degrading proteins within mammalian cells (Glickman and Ciechanover, 2002). The UPS is comprised of a network of signaling molecules that identify, tag, and degrade substrate proteins within the cell (Figure 2). In this system, proteins are first targeted for degradation by the covalent attachment of the small protein modifier ubiquitin *via* the coordinated actions of three separate classes of ubiquitin ligases (E1, E2, and E3). The activating enzyme, E1, binds to and activates free ubiquitin in an ATP-dependent reaction. E1 then transfers activated ubiquitin to an E2 ligase that carries the active ubiquitin to the substrate protein. The substrate proteins to be degraded are recognized by specific E3 ligases which identify degradation signals emitted by the substrate proteins themselves (Nandi et al., 2006). The E2 ligase then binds to the E3-substrate complex, enabling the transfer of activated ubiquitin to the substrate protein (Figure 2A). The ubiquitination process is highly complex and involves hundreds of different ligases that interact in a combinatorial manner to achieve substrate specificity. In the human genome, the coding genes for each ligase total 1–2 for E1, 25–30 for E2, and more than 600 for E3. Although substrate specificity is primarily achieved by the vast number of E3 ligases, specificity is also achieved by limited interactions of E2-E3 proteins. For example, E2s bind to numerous different E3s, but not every E3 can interact with every E2. Therefore, the E2s, E3s, and substrate proteins come together to create a unique combinatorial code for the ubiquitin reaction (Hegde, 2017). After the first ubiquitin is bound to the substrate protein, another ubiquitin becomes attached to an internal lysine residue on the first ubiquitin, eventually forming a polyubiquitin chain. Substrate proteins can acquire several different types of ubiquitin “tags,” however, those that receive a lysine-48 (K48) polyubiquitin tag become targets for degradation by the 26S proteasome complex (Glickman and Ciechanover, 2002; Musaus et al., 2020).

The 26S proteasome is a multi-subunit structure comprised of a cylindrical 20S core particle (CP) flanked by one or two 19S regulatory particles (RP; Tanaka, 2009). The 19S RP contains an outer lid comprised of a circular ring of non-ATPase subunits where polyubiquitinated protein initially binds (Figure 2B). When a polyubiquitinated substrate is bound to the outer segment of the 19S CP, the polyubiquitinated chain becomes hydrolyzed by deubiquitinating enzymes so that the ubiquitin molecules can be reused in the system. The 19S RP also contains an inner cap segment that consists of a circular ring of six ATPase subunits that, when activated, are responsible for initiating unfolding and translocating the protein into the catalytic 20S core of the proteasome. The 20S CP is comprised of two outer rings of α-subunits and two inner rings of β-subunits. The outer α-subunits are connected to the inner ATPase subunits of the 19S cap which gives the proteasome its gate-like mechanism of action. When the 19S ATPase subunits become activated, the α-subunits enable the substrate to pass through its gated channel. However, when a polyubiquitinated substrate is not bound to the proteasome, the α-subunit gate remains closed to prevent the degradation of intact protein, as well as the release of partially degraded substrate protein from the 20S CP. The substrate is then degraded by various catalytic activities (i.e., chymotrypsin-like, trypsin-like, and caspase-like activity) of the 20S CP, which are exerted by the inner β5, β2, and β1 subunits, respectively. The resulting peptide fragments are then expelled through the base of the proteasome.

### Regulation of Proteasome Subunits by PKA and CaMKII

As illustrated in Figure 2, proteasomes are multi-subunit complexes that must be assembled to exert their chymotrypsin-like, trypsin-like, and caspase-like proteolytic activities. Proteasome subunit phosphorylation is a principal mechanism that regulates proteolysis by altering proteasome assembly, localization, or its catalytic activity (Hegde, 2004; Nandi et al., 2006). Phosphorylation of the 19S *r*egulatory *p*article *t*riple-ATPase *6* subunit at the serine 120 residue (Rpt6 at Ser^120^, hereafter referred to as Rpt6; Figure 2B) is the most commonly studied phosphorylation site in the context of synaptic plasticity and memory because this subunit is targeted by PKA and CaMKII. Because these kinases are regulated by E_2_, they may mediate its effects on UPS signaling. As such, the regulation of UPS function by PKA and CaMKII will be discussed below.

Thus far, evidence to show that PKA regulates proteasome activity comes from studies of non-neuronal cells or tissues not typically associated with memory. Forskolin, a compound that elevates cyclic AMP (cAMP) levels and activates PKA, stimulates chymotrypsin-like and trypsin-like peptidase activity in nuclear extracts from cultured normal rat kidney cells by phosphorylating Rpt6 (Zhang et al., 2007). Moreover, this forskolin-induced increase in proteasome activity could be blocked pharmacologically, indicating that PKA-dependent phosphorylation is responsible for the increases in peptidase activity (Zhang et al., 2007). Phosphorylation of Rpt6 by the cAMP/PKA pathway is also critical in regulating the neuropathology of Huntington’s disease. For example, striatal cells expressing mutant Huntington protein have markedly low PKA activity that prevents phosphorylation of Rpt6 (Lin et al., 2013). Expression of phosphomimetic Rpt6 rescued motor impairments and reduced mutant Huntington protein aggregates in the striatal synaptosome fraction from Huntington’s mice (Lin et al., 2013), suggesting an important role for Rpt6 in both pathology and behavior. Additional evidence suggests that PKA regulates proteasome activity by increasing transcriptional levels of proteasome subunits. Artificially increasing cAMP levels in rat spinal cord neurons not only increases chymotrypsin-like activity but also increases mRNA and protein levels of Rpt6 and the 20S proteasome subunit β5 in a PKA-dependent manner (Myeku et al., 2012).

Rpt6 can also be phosphorylated by CaMKIIα in a neuronal activity-dependent manner (Bingol et al., 2010), which then leads to proteasome trafficking into dendritic spines. In cultured hippocampal neurons, synaptic activity causes autophosphorylated CaMKIIα to act as a postsynaptic scaffolding molecule by physically binding to 19S and 20S proteasome complexes and translocating them from dendritic shafts to dendritic spines, where they co-localize to the actin cytoskeleton in an NMDAR-dependent manner (Bingol and Schuman, 2006; Bingol et al., 2010). These findings are consistent with other work demonstrating that proteasome activity in dendrites of cultured hippocampal neurons is regulated by synaptic activity and that overexpression of a constitutively active form of CaMKII activates proteasomes by phosphorylating Rpt6 (Djakovic et al., 2009). Accordingly, homeostatic scaling of synaptic strength in rodent hippocampal slices was impaired by altered CaMKII-dependent phosphorylation of Rpt6 (Djakovic et al., 2012). CaMKII-induced phosphorylation of Rpt6 also regulates hippocampal spinogenesis, as expression of a phospho-dead mutant of Rpt6 prevented activity-induced hippocampal spine outgrowth on postsynaptic neurons (Hamilton et al., 2012). Furthermore, Rpt6 phosphorylation is also increased in the amygdala of male rats following contextual fear conditioning (Jarome et al., 2013), suggesting that activity-induced Rpt6 phosphorylation may promote the structural plasticity underlying memory formation. Interestingly, the learning-induced increase in Rpt6 phosphorylation depends on CaMKIIα, but not PKA, activity in male rat amygdala tissue (Jarome et al., 2013), indicating greater involvement of CaMKII in mediating the proteasome activity stimulated by learning. It should be noted that one recent study using knock-in mouse models to block or mimic Rpt6 activity indicated no involvement in measures of plasticity, spine growth, or fear conditioning (Scudder et al., 2021), however, compensatory mechanisms may have mitigated the loss of a functional Rpt6 Ser^120^ subunit (Lokireddy et al., 2015; Guo et al., 2016). Overall, these findings suggest that CaMKIIα-dependent phosphorylation of Rpt6 may be critical for regulating the proteasomal protein degradation involved in synaptic remodeling and long-term memory.

## Protein Degradation and Synaptic Plasticity

Substantial evidence supports a role for protein degradation as a critical regulator of activity-dependent synaptic plasticity (Fioravante and Byrne, 2010; Jarome and Helmstetter, 2013; Hegde, 2017). Initial studies conducted in *Aplysia* investigated how cAMP-dependent PKA remained persistently active following long-term facilitation (LTF) when levels of cAMP were depleted. This work revealed that the increased pool of PKA regulatory subunits became ubiquitinated and degraded by the proteasome, leaving the catalytic subunits intact and persistently active (Hegde et al., 1993). Additional work in *Aplysia* demonstrated that LTF increased expression of the ubiquitin C-terminal hydrolase (Ap-uch), which is responsible for deubiquitinating proteasome-bound protein, thereby increasing proteasome activity within neurons. Inhibiting the expression of Ap-uch blocked the induction of LTF (Hegde et al., 1997). Similarly, the injection of proteasome inhibitors prevented induction of LTF in *Aplysia* (Chain et al., 1999).

Later studies in hippocampal slices from male rats examined the extent to which the proteasome regulates protein-synthesis-dependent late-phase LTP (L-LTP). L-LTP is comprised of an induction phase that requires translation of pre-existing mRNAs at dendrites, and a maintenance phase that requires *de novo* transcription in the nucleus (Kelleher et al., 2004). In hippocampal slices, L-LTP can be induced by forskolin, thereby supporting a key role for PKA. Interestingly, proteasome inhibition has different effects on forskolin-induced L-LTP based on the location of proteasome inhibition. For example, proteasome inhibition at dendrites enhanced the induction phase of L-LTP, whereas proteasome inhibition at the nucleus blocked the maintenance phase of L-LTP (Dong et al., 2008). These findings led to speculation that proteasome inhibition enhances L-LTP induction by preventing the degradation of proteins synthesized from pre-existing mRNAs, but blocks L-LTP maintenance by preventing the degradation of transcriptional repressors in the nucleus. Subsequent work supported this hypothesis, as inhibition of ERK and mTORC1 signaling in dendrites prevented proteasome inhibition from enhancing the induction phase of L-LTP (Dong et al., 2014). These novel findings showed that the proteasome can paradoxically control local protein translation by regulating the activity of translational activators and repressors throughout the induction and maintenance phases of L-LTP.

Other studies have assessed the effects of proteasome inhibition on L-LTP and found that the maintenance phase, but not the induction phase, of L-LTP was blocked entirely in hippocampal slices from male rats when either protein synthesis or protein degradation was inhibited. These impairments were rescued if protein synthesis and protein degradation inhibitors were applied at the same time (Fonseca et al., 2006; Karpova et al., 2006). These findings conflict with the previous reports discussed above that proteasome inhibition enhanced the induction phase of L-LTP (Dong et al., 2008, 2014), which could result from their use of a lower proteasome inhibitor dose or a less-specific proteasome inhibitor. Nonetheless, these findings collectively demonstrate that a delicate balance between protein synthesis and protein degradation must exist to support L-LTP and maintain long-lasting synaptic plasticity.

This work also began to illustrate that the proteasome regulates hippocampal synaptic plasticity by targeting diverse substrate proteins throughout neuronal synaptic and nuclear compartments (Hegde et al., 2014). For example, the activity-induced translocation of proteasomes to dendritic spines causes other structural changes that influence synaptic plasticity. Synaptic activity in bicuculline-treated cortical neurons significantly increases the number of ubiquitinated proteins in the synapse and PSD (Ehlers, 2003). In particular, the post-synaptic scaffolding proteins Shank, GKAP, and AKAP79 are ubiquitinated and degraded in response to synaptic activity, an effect that is abolished following periods of inactivity (Ehlers, 2003). These cytoskeletal and scaffolding proteins become targets for proteasomal degradation following synaptic activity because they directly support glutamate receptors within the PSD (Sheng and Pak, 2000; Sheng and Kim, 2002). AMPA receptor internalization following NMDAR activation in hippocampal neurons depends on the ubiquitination and subsequent degradation of PSD-95 (Colledge et al., 2003; Patrick et al., 2003; Bingol and Schuman, 2004). Protein degradation also appears to regulate spine shape, as the activity-inducible kinase SNK is targeted to dendritic spines and is responsible for initiating the degradation of the PSD protein SPAR (Pak and Sheng, 2003). SPAR was also shown to be degraded by the proteasome in an NMDA-dependent manner following LTP induction in CA1 neurons from male rats (Chen et al., 2012). As such, UPS activity provides localized protein degradation within synapses to rapidly remodel the spine morphology that supports enhanced plasticity.

Finally, ubiquitin-mediated proteasomal protein degradation can also influence plasticity by degrading nuclear proteins that inhibit gene transcription. For example, induction of LTF in *Aplysia* depends on the ubiquitination and proteasomal degradation of the cAMP-response element binding protein (CREB) repressor protein activating transcription factor 4 (ATF4; Upadhya et al., 2004). Furthermore, proteasome inhibition following chemically-induced LTP prevents the ubiquitination and degradation of ATF4, effectively preventing the transcription of CREB-inducible genes, such as brain-derived neurotrophic factor (*bdnf*, Dong et al., 2008). More recent work has shown that the E3 ligase β-transducin repeat containing protein (β-TrCP) ubiquitinates ATF4 in a PKA-dependent manner (Smith et al., 2020). Therefore, the proteasome not only serves to degrade synaptic proteins but also to regulate gene expression by degrading machinery that exerts repressive effects on the transcription of genes critical for synaptic plasticity and memory.

## Protein Degradation and Long-Term Memory

Numerous studies have demonstrated that proteasomal protein degradation is not only critical for activity-dependent synaptic plasticity but also plays an important role in regulating long-term memory. These studies have predominantly used proteasome inhibitors such as lactacystin (lac) and clasto-lactacystin β-lactone (β-lac) to block the catalytic activities of the proteasome (Ōmura and Crump, 2019). Pharmacological blockade of proteasome activity has enabled researchers to examine the extent to which proteasome activity is required for the consolidation and reconsolidation phases of memory storage. The behavioral work to date has primarily examined the involvement of protein degradation in fear learning among male rats (for comprehensive reviews, see Jarome and Helmstetter, 2013, 2014; Hegde, 2017). However, recently published data suggest differences in how males and females regulate and engage in proteasome activity, which will be discussed in more detail below.

Initial work demonstrated that immediate post-training bilateral infusion of lac into the CA1 region of the DH caused full retrograde amnesia for one-trial inhibitory avoidance learning in male rats (Lopez-Salon et al., 2001). Subsequently, bilateral infusion of β-lac into CA1 was shown to impair the extinction, but not consolidation or reconsolidation, of contextual fear memory in male rats (Lee et al., 2008), suggesting a potentially complex role for CA1 protein degradation in fear learning. Protein degradation in other brain regions also contributes to fear learning, as bilateral infusion of lac in the amygdala and insular cortex impaired consolidation of conditioned taste aversion memories (Rodriguez-Ortiz et al., 2011). Moreover, immediate post-training infusion of β-lac into the amygdala impaired the consolidation of both auditory and contextual fear memories (Jarome et al., 2011). Although the same study observed NMDA-dependent increases in polyubiquitination of RISC factor MOV10 and scaffolding protein Shank, β-lac infusion into the amygdala following memory retrieval did not impair auditory or contextual fear memory reconsolidation, but did rescue impairments caused by infusion of protein synthesis inhibitor anisomycin (Jarome et al., 2011), suggesting that protein degradation controls destabilization of retrieved fear memories in the amygdala. Subsequent findings also demonstrated that β-lac in male rats impaired trace fear conditioning when infused into the prefrontal cortex, and impaired contextual memory in a context-preexposure facilitation paradigm when infused into the dorsal and ventral hippocampus (Reis et al., 2013; Cullen et al., 2017). Interestingly, recent work investigating AMPA receptor (AMPAR) exchange at synapses in the amygdala of male rats demonstrated that proteasome activity is critical for the endocytosis of calcium-impermeable AMPARs with calcium-permeable AMPARs during the destabilization phase of reconsolidation (Ferrara et al., 2019). Together, these data suggest an important role for UPS activity in numerous brain regions in mediating fear learning among male rats.

A requirement for proteasomal protein degradation has also become evident for spatial and object recognition memories. Infusion of lac into the hippocampal CA3 subregion of male mice significantly impaired spatial memory consolidation and reconsolidation in Morris water maze when infused immediately, but not 3 h, post-training (Artinian et al., 2008). These findings provided the first demonstration that different phases of non-aversive memory formation require proteasomal protein degradation. Consistent with this conclusion, another study utilizing an object rearrangement task in male mice assessed whether proteasome activity is required for incorporating partially modified information into a pre-existing memory, and found that infusions of β-lac into area CA1 following re-exposure to the context with switched objects disrupted the initial consolidation of spatial information (Choi et al., 2010). Similarly, object recognition memory consolidation in male rats was disrupted by proteasome inhibition, as the infusion of lac into CA1 immediately and 3 h, but not 1.5 or 6 h, post-training significantly reduces the time spent with a novel object during testing (Figueiredo et al., 2015). However, these findings are inconsistent with other work demonstrating that post-training infusion of β-lac into CA1 did not impair consolidation or reconsolidation of object recognition memory in male rats (Furini et al., 2015). Interestingly, however, β-lac infusion reversed reconsolidation impairments caused by anisomycin (Furini et al., 2015). The discrepancies between this and the Figueiredo et al. (2015) study could result from the administration of different proteasome inhibitors at different doses. Nevertheless, the balance of studies conducted so far suggests a potential role for hippocampal proteasomal protein degradation in spatial and object memories among males.

Collectively, these studies suggest that protein degradation mediated by the UPS is essential for different forms of learning across numerous brain regions. Moreover, UPS activity and hippocampal synaptic plasticity are regulated by protein kinases that are also involved in E_2_’s effects on memory consolidation. This overlap suggests compelling reasons to suspect that E_2_’s well-documented effects on spatial and object recognition memory consolidation might be regulated in part by proteasomal protein degradation.

## Emerging Sex Differences in UPS Activity and Memory

Several recent studies have documented notable sex differences in the regulation of, and requirement for, protein degradation following fear memory formation in the basolateral amygdala (BLA) and CA1 region of the DH (Devulapalli et al., 2019, 2021; Martin et al., 2021). This work has also revealed novel sex differences in the number and identity of substrate proteins targeted for proteasomal degradation across BLA and DH tissues (Farrell et al., 2021; Martin et al., 2021).

The first study to examine putative sex differences in UPS activity related to memory showed that CaMKII and PKA differentially regulate proteasome activity in male and female rats across subcellular compartments following contextual fear conditioning (Devulapalli et al., 2019). Tissue in these studies was fractionated to isolate synaptic, cytosolic, nuclear compartments. Chymotrypsin activity, the predominant form of proteasome activity, was decreased in synaptic fractions following CaMKII, but not PKA, inhibition in the male DH, whereas chymotrypsin activity was increased in synapses following CaMKII, but not PKA, inhibition in the female DH. These data suggest that proteasome activity is not only differentially regulated CaMKII and PKA activity across subcellular compartments but is also regulated in a sex-specific manner. Moreover, the regulatory effects of CaMKII and PKA also differed across brain regions. For example, nuclear chymotrypsin activity was decreased following PKA, but not CaMKII, inhibition in the male BLA, whereas nuclear chymotrypsin activity was decreased following CaMKII, but not PKA, inhibition in the female BLA (Devulapalli et al., 2019). These findings are noteworthy because they not only provide support for the idea that proteasome activity can be differentially regulated across subcellular compartments (Upadhya et al., 2006) but also highlight the differences that exist between males and females in the regulation of proteasome function by signaling kinases.

More recently, males and females were found to differ in their engagement and requirement for UPS activity following contextual fear conditioning. For example, trained male, but not female, rats exhibited increased markers of UPS activity, including upregulated proteasome activity and amount of K48 polyubiquitinated proteins, in nuclear BLA extracts relative to behaviorally naïve males (Devulapalli et al., 2021). Interestingly, both naïve and trained females displayed elevated UPS activity relative to naïve males, suggesting higher baseline levels of UPS activity in nuclear BLA extracts among females relative to males (Devulapalli et al., 2021). This finding could have indicated that learning does not engage the UPS in females, however, CRISPR-dCas9-mediated knockdown of UPS activity in BLA was found to impair fear memory in both sexes (Devulapalli et al., 2021), suggesting that males and females differ in their engagement, but not requirement for, UPS activity in the BLA for successful fear memory formation. This conclusion was supported by additional data showing that female rats had elevated levels of free ubiquitin and increased expression of the ubiquitin coding gene *Uba52* in BLA nuclear extracts relative to males (Devulapalli et al., 2021), indicating inherently higher numbers of ubiquitinated targets in females relative to males. Furthermore, naïve female rats exhibited increased 5-hydroxymethylation in the promoter region of the ubiquitin coding gene *Uba52*, suggesting that this gene is more actively transcribed in females than in males. Nevertheless, CRISPR-dCas9-mediated silencing of the ubiquitin coding gene *Uba52* and the proteasome subunit *Psmd14* in the BLA of male and female rats reduced baseline protein degradation levels and impaired contextual fear memory, whereas increasing BLA baseline protein degradation facilitated fear memory in both sexes (Devulapalli et al., 2021). Thus, despite sex differences in baseline ubiquitination in the BLA, fear memory formation in both males and females appears to depend on UPS activity.

Surprisingly, the sex-specific activation of UPS activity by contextual fear conditioning differs strikingly in the DH. In DH nuclear extracts, learning-induced increases in UPS activity were observed in female, but not male, rats (Martin et al., 2021). Moreover, CRISPR-mediated knockdown of UPS activity in DH CA1 blocked fear memory in females, but not males (Martin et al., 2021). These data suggest that females require UPS activity in the DH to form a contextual fear memory, whereas males do not, which contrasts with the BLA in which both sexes require UPS activity for memory formation. Thus, for fear learning, the involvement of protein degradation appears to differ not only by sex by also by brain region across the fear circuit.

Other recent work examined sex differences in UPS activity at 3, 15, and 22 months of age in response to trace fear conditioning to a tone. Age-related memory impairments in trace fear retrieval in male, but not female rats were associated with decreased Rpt6 phosphorylation and increased K48 polyubiquitination in synaptic fractions of BLA tissue (Dulka et al., 2021). Specifically, 22-month-old male rats exhibited impaired memory retrieval 24 h after training, whereas females of all ages displayed relatively poor retrieval at all ages. Among male rats, retrieval-induced Rpt6 phosphorylation was significantly reduced in 22-month-olds relative to 3-month-olds in the BLA, but no changes were observed in the DH or medial prefrontal cortex. Interestingly, 22-month-old females exhibited lower Rpt6 phosphorylation in the cortex relative to 3-month-olds, but no retrieval-induced changes in the other two brain regions. With respect to K48 polyubiquitination, a similar regional pattern was observed, with 22-month-old males having increased levels in the BLA, whereas females had higher levels in the cortex. suggesting that the memory impairments observed in aged males may arise in part by dysregulated proteasome signaling that results in an accumulation of polyubiquitinated substrate proteins. Together, these findings suggest that the role of UPS activity in memory may differ not only by sex and brain region but by age as well.

To date, most studies examining ubiquitin-proteasome function in the context of learning and memory have examined the factors that regulate proteasome activity itself, leaving unanswered questions about which proteins are targeted for proteasomal degradation following learning. Exciting new work sheds light on the number and identity of protein substrates targeted for proteasomal protein degradation following contextual fear conditioning in both sexes. These studies used an unbiased assay that focuses on K48-specific ubiquitination because this particular lysine tag marks proteins for degradation. This novel K48-specific tandem ubiquitin binding entity (K48-TUBE) liquid chromatography-mass spectrometry analysis captures K48-polyubiquitnated proteins with high affinity, thereby protecting them from proteasomal degradation and deubiquitination, permitting efficient purification, and eliminating non-specific binding. This method has revealed that the number of proteins in the BLA in which K48 polyubiquitination was increased or decreased in response to fear learning overlaps very little between males and females (Farrell et al., 2021). Interestingly, fear learning promoted protein degradation in both sexes, but more so in females, which is in contrast to previous reports that nuclear UPS activity in the BLA was not increased by fear conditioning (Devulapalli et al., 2021); these discrepancies likely result from the increased sensitivity and specificity of the K48-TUBE assay to detect polyubiquitinated proteins relative to immunoblotting (Farrell et al., 2021). In the DH, contextual fear conditioning increased K48 polyubiquitin targeting of only three protein targets in the CA1 of females, whereas learning did not increase K48 polyubiquitination of any proteins in males (Martin et al., 2021). This result is perhaps surprising but is consistent with the finding that contextual fear conditioning did not increase UPS activity in the CA1 of males (Martin et al., 2021). An ingenuity pathway analysis of proteins in the female CA1 showed that fear learning targets the ribosomal binding protein ribosomal RNA processing 12 (RRP12) and chaperone protein heat shock protein 40 (HSP40) for degradation, which has implications for the regulation of intracellular signaling and DNA damage response (Martin et al., 2021). Collectively, these initial findings indicate little overlap between the sexes in how learning influences the targeting of proteins for proteasomal protein degradation in the BLA and CA1 and suggest that a variety of different cellular processes are regulated in a sex-, brain region-, and degradation-specific manner to support the formation of fear memories.

These recent studies not only uncover key sex differences in the regulation of, and requirement for, ubiquitin-proteasome activity in different brain regions for the formation of fear memories, but also reveal sex differences in the number and identity of proteins targeted for proteasomal degradation following learning. It is tempting to speculate, therefore, that sex steroid hormones, such as E_2_, play a major role in these effects, although this hypothesis has not yet been tested. In the next sections, we discuss evidence that E_2_ can regulate UPS activity and highlight commonalities between E_2_- and UPS-signaling that support a potential role for UPS activity in the mnemonic effects of E_2_.

## Estrogenic Regulation of UPS Activity

The data discussed thus far support the conclusion that the UPS is not only involved in regulating synaptic plasticity and long-term memory but also exerts its proteolytic effects to support memory formation in a sex-specific manner. Although not yet examined in the context of learning and memory, evidence also suggests that the UPS can be directly stimulated by E_2_.

Data from non-neuronal cells indicate some reciprocal interactions between E_2_ and UPS activity. ERα and ERβ are rapidly degraded by the proteasome after they translocate to the nucleus and bind to estrogen response elements on target gene promoters to activate or repress gene transcription (Zhou and Slingerland, 2014; Kondakova et al., 2020). In HeLa cells, estrogen receptors are degraded by the proteasome in an E_2_-dependent manner, as the application of proteasome inhibitors MG132 or lactacystin increased ER levels by blocking E_2_-induced ER degradation (Nawaz et al., 1999). Additionally, E3 ligases appear to act as transcriptional co-activators for ERs, where they are uniquely positioned to rapidly ubiquitinate E_2_-bound ERs for degradation (Shang et al., 2000). Although these data were collected from *in vitro* work assessing breast and endometrial cancers, they lend support to the possibility that E_2_ might recruit UPS activity through canonical signaling pathways to promote hippocampal memory formation. This possibility is buoyed by findings showing that E_2_ can stimulate the UPS by rapidly activating cell-signaling mechanisms. For example, E_2_ causes ERK-dependent phosphorylation of the cyclin-dependent kinase inhibitor p27, which results in the increased ubiquitination and proteasomal degradation of p27, and subsequent unchecked proliferation of endometrial epithelial cells (Lecanda et al., 2007; Huang et al., 2012).

Limited evidence also suggests that E2 signaling in the hippocampus and cortex can directly stimulate UPS activity. For instance, treatment of hippocampal slices with E_2_ increased ubiquitination and proteasomal-mediated degradation of GluA1-containing AMPA receptors in the CA3 region of the male rat hippocampus (Briz et al., 2015). Other work has shown that ERα in rat hippocampal CA1 undergoes enhanced proteasomal degradation following long-term E_2_ deprivation, an effect that was prevented when E_2_ was administered before, but not after, E_2_ deprivation (Zhang et al., 2011). A separate study in cultured primary cortical neurons found that Cav1.2, a pore-forming subunit of L-type voltage gated calcium channel, is ubiquitinated by the E3 ligase Mdm2 and degraded by the proteasome in an ERα-dependent manner (Lai et al., 2019). Moreover, this study demonstrated in an ovariectomized Alzheimer’s mouse model that systemic administration of an ERα agonist, but not ERβ agonist, reduced Cav1.2 protein in the hippocampus and cortex by increasing ubiquitination and subsequent degradation of Cav1.2 by Mdm2 (Lai et al., 2019). Thus, although relatively scant, there is some basis on which to speculate that E_2_ and the ERs may regulate UPS activity in cognitive brain regions such as the hippocampus and cortex and that the resulting protein degradation may influence memory formation.

## Overlapping Mechanisms in E_2_- and UPS-Signaling

E_2_ facilitates hippocampal spine density and memory consolidation in both males and females by interacting with receptors positioned at the plasma membrane to promote a cascade of rapid cell signaling events that regulate protein synthesis to support synaptic plasticity (Frick, 2015; Taxier et al., 2020). Interestingly, the signaling events so far identified as critical for regulating E_2_’s effects on structural plasticity and memory overlap considerably with those that enable UPS to regulate synaptic plasticity and memory.

For example, E_2_ promotes NMDAR signaling by increasing excitatory postsynaptic potential amplitude and receptor binding, and increases hippocampal sensitivity to NMDAR inputs (Woolley et al., 1997; Foy et al., 1999). NMDAR activation is required for many of E_2_’s effects, including enhanced LTP (Foy et al., 1999), dendritic spine density (Woolley and McEwen, 1994), and hippocampus-dependent memory (Lewis et al., 2008; Vedder et al., 2013). Our lab has shown that DH infusion of an NMDAR antagonist prevents E_2_ from enhancing object recognition memory and activating DH cell signaling in ovariectomized mice (Lewis et al., 2008), suggesting that NMDA activity is necessary for E_2_ to facilitate memory formation. Similarly, NMDAR activity is required for male rats to increase the amount of polyubiquitinated proteins in the amygdala following auditory fear retrieval (Jarome et al., 2011). Additionally, NMDAR activity is required for proteasomes to be redistributed to hippocampal dendritic spines (Bingol and Schuman, 2006; Ferreira et al., 2021) and for targeting polyubiquitination of synaptic scaffold proteins (Colledge et al., 2003; Guo and Wang, 2007).

In addition to having similar requirements for NMDAR activity, E_2_ and the UPS both increase the activity of CaMKII and PKA to exert their beneficial effects on memory. For example, systemic administration of E_2_ rapidly increases phosphorylation of CaMKII in ovariectomized mice, a molecular effect that depends on the activation of estrogen receptors (Sawai et al., 2002). Similarly, calcium influx through NMDARs increases CaMKII phosphorylation, which then phosphorylates Rpt6 (Djakovic et al., 2009, 2012), thereby increasing proteasomal activity and proteasome redistribution to synapses (Bingol et al., 2010). CaMKII-mediated phosphorylation of Rpt6 drives hippocampal dendritic spine outgrowth (Hamilton et al., 2012) and fear memory formation in male rats (Jarome et al., 2013). Furthermore, work from our lab demonstrates that E_2_ requires PKA activity to enhance object recognition memory consolidation in ovariectomized female mice (Lewis et al., 2008). Other findings show that E_2_ requires PKA activity to potentiate synapses in hippocampal slices from female, but not male, rats (Jain et al., 2019). A sex-specific role for PKA activity also appears to be critical for regulating proteasome activity across subcellular compartments of DH and BLA neurons following contextual fear conditioning (Devulapalli et al., 2019). Thus, there are several overlapping mechanisms through which E_2_ and the UPS regulate synaptic plasticity and long-term memory which provide support for the possibility that E_2_ might require aspects of UPS signaling to exert its neuromodulatory effects.

Although the similarities in the requirements for NMDAR, CaMKII, and PKA activity for both E_2_ and the UPS provide compelling reasons to suspect UPS involvement in E_2_’s ability to facilitate memory consolidation, additional support for this hypothesis comes from the notion that the successful formation long-term memories requires a delicate balance between protein synthesis and protein degradation (Park and Kaang, 2019). Evidence of the involvement of both processes can be seen at the synaptic level, where polyribosomes are transported to dendritic spines to promote local protein synthesis (Bramham and Wells, 2007) at the same time that proteasomes are being translocated to dendrites to promote local protein degradation of synaptic scaffolding molecules (Bingol and Schuman, 2006; Shen et al., 2007; Bingol et al., 2010). Similarly, at the behavioral level, expression levels of mTOR and its downstream effector p70S6 kinase were significantly increased at the same time that levels of K48 polyubiquitination were increased in the amygdala of male rats 1 h following contextual fear conditioning (Jarome et al., 2011). E_2_ acts at membrane-localized ERs in the DH to activate mTORC1 signaling, which is necessary for E_2_ to increase CA1 dendritic spine density in the DH and enhance the spatial and object recognition memory consolidation in ovariectomized mice (Fortress et al., 2013; Tuscher et al., 2016). Therefore, one might suspect that activity-dependent increases in protein synthesis would precipitate similar increases in the opposite, but equally important process, of protein degradation.

## Hypothesized Mechanism of Estrogenic Regulation of UPS

**Figure 3:**
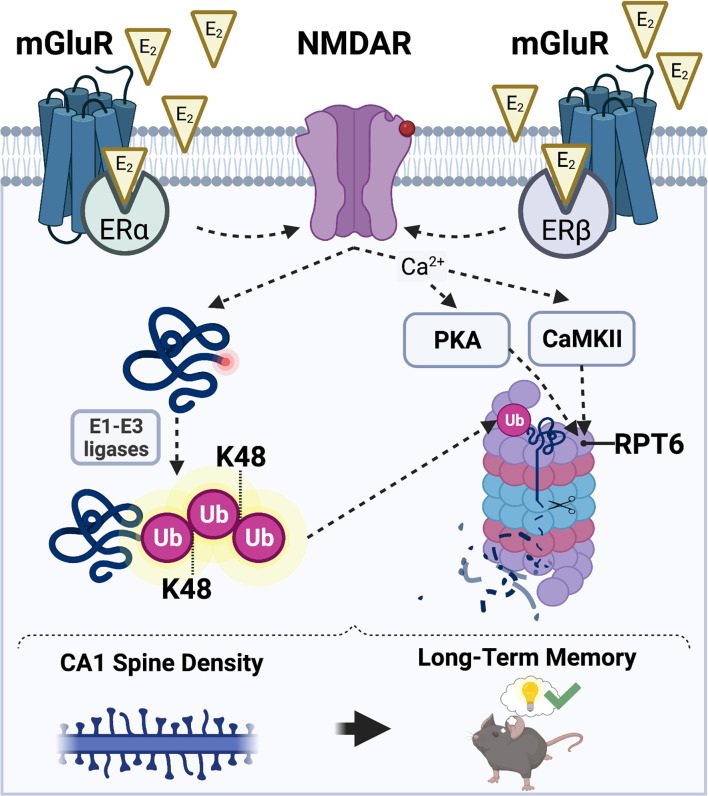
Hypothesized mechanisms through which E_2_ may activate the UPS to regulate CA1 dendritic spine density and hippocampal memory. E_2_ acts *via* membrane-associated estrogen receptors including ERα and ERβ to promote postsynaptic sensitivity to glutamate (red circle on NMDAR) and opening of NMDARs. E_2_-induced NMDAR activation promotes an increase in the amount of substrate proteins that acquire a K48-polyubiquitin tag through the actions of E1-E3 ubiquitin ligases (left). NMDAR activation also simultaneously permits an influx of intracellular Ca^2+^ which results in an E_2_-induced increase in CaMKII and PKA activity (right). CaMKII and PKA then phosphorylate the Rpt6 subunit of the 26S proteasome complex, which mobilizes proteasomes to dendritic spine shafts to initiate the breakdown of K48-tagged substrate proteins. Illustration created using BioRender.com.

Based on the literature reviewed above, we hypothesize that E_2_ may promote CA1 spine density and hippocampal memory formation in males and females by increasing UPS activity, which would cause the degradation of structural proteins localized in the PSD to allow for synaptic remodeling in response to a learning event. In our model of E_2_-induced activation of UPS signaling (Figure 3), we propose that E2 stimulates UPS activity by binding to membrane-associated ERα and ERβ which then increase NMDAR activity. E_2_-induced activation of NMDARs could trigger an increase in UPS activity by: (1) upregulating the amount of K48-linked polyubiquitinated proteins in the synapse through the actions of E1-E3 ubiquitin ligases; and (2) increasing the assembly and localization of 26S proteasomes to synapses by CaMKII- and PKA-dependent phosphorylation of the Rpt6 26S proteasome subunit. However, it is important to note that the nature of the interaction between E_2_ and hippocampal NMDAR activation in males and females remains unclear. Although we speculate that E_2_ activates NMDARs through interaction with membrane-associated ERs, there are alternative putative mechanisms through which NMDARs may be activated by E_2_ to initiate UPS signaling. For example, E_2_ may indirectly increase NMDAR activity by regulating the activity of AMPAR-mediated currents (Srivastava et al., 2008; Smejkalova and Woolley, 2010; Jain et al., 2019). This E_2_-induced activation of AMPARs could theoretically regulate NMDAR activity and, thereby, calcium influx and downstream activation CaMKII and PKA, to facilitate both protein synthesis and protein degradation in ways that increase spine density.

We hypothesize that E_2_ promotes local protein degradation in a rapid manner that coincides with the need for local protein synthesis. We have previously documented E_2_-induced and mTOR-dependent increases in CA1 spine density in ovariectomized mice 30 min after DH infusion (Tuscher et al., 2016), and other reports show that DH infusion of E_2_ or the GPER agonist G-1 increases CA1 spine density in ovariectomized mice within 40 min (Phan et al., 2015; Kim et al., 2019). Because UPS activity relies on many of the same signaling pathways as E_2_-induced memory enhancement and spinogenesis, it is plausible that E_2_ could increase UPS activity as soon as 30 min following DH infusion. Nevertheless, such rapid action would not exclude the possibility of more long-term activation *via* genomic or epigenomic actions of ERα and ERβ.

We speculate an involvement of ERα and ERβ because work from our lab and others indicates that ERα and ERβ agonism facilitates the consolidation of object recognition and spatial memories in ovariectomized rats and mice (Jacome et al., 2010; Kim and Frick, 2017; Hanson et al., 2018; Fleischer et al., 2021). Moreover, ERα and ERβ in the DH interact directly with mGluR1a to trigger the ERK signaling that is necessary for object placement and object recognition memory consolidation in ovariectomized mice (Boulware et al., 2013), which suggests that these receptors both influence memory at the plasma membrane to promote hippocampal memory formation. Although both ERα and ERβ play discrete roles in regulating synaptic potentiation in male and female rats (Kramár et al., 2009; Smejkalova and Woolley, 2010; Oberlander and Woolley, 2016), the interaction between E_2_ and NMDARs may be particularly mediated by ERα, whose agonism has been shown to increase the expression of NMDARs in the DH of ovariectomized rats (Morissette et al., 2008). ERα agonism has also been shown to increase NMDAR-mediated EPSCs and lower the threshold for the induction of NMDA-dependent LTP in the dentate gyrus of male rats (Tanaka and Sokabe, 2013). Thus, ERα may play a larger role in activating UPS signaling than ERβ.

Although we have proposed that UPS-mediated protein degradation is required for the E_2_-induced facilitation of CA1 spine density and memory consolidation in both sexes, we suspect that the signaling mechanisms that regulate this activity may differ considerably between males and females. For example, although E_2_ can increase synaptic potentiation in both sexes, males and females utilize different ERs at pre- and post-synaptic sites to facilitate synaptic potentiation (Oberlander and Woolley, 2016). In males, presynaptic increase in glutamate release is mediated by ERα and postsynaptic increase in glutamate sensitivity is mediated by ERβ. However, in females, the presynaptic increase in glutamate release is mediated by ERβ and the postsynaptic increase in sensitivity is mediated by GPER (Oberlander and Woolley, 2016). Based on these findings, we speculate that the effects of E_2_ on UPS activity in males and females may be regulated in part by different ERs. Furthermore, sex differences may also exist in the signaling mechanisms that occur downstream of our proposed model of E_2_-induced NMDAR activation. We speculate that E_2_ increases CaMKII and PKA activity in a manner that is dependent on ER-driven activation of NMDARs. There is sufficient evidence to suggest that males and females might differ in their requirements for CaMKII and PKA activity to initiate UPS activity following E_2_ exposure. For example, PKA is required for acute E_2_-induced initiation of synaptic potentiation in females, but not males (Jain et al., 2019). These findings potentially suggest that PKA activity may be required for E_2_ to increase UPS activity in females, but not males, at hippocampal synapses. In males, E_2_-mediated UPS activity could be more driven by CaMKII or K48 polyubiquitination.

In sum, our hypothesis posits multiple possible mechanisms through which E_2_ might activate the UPS system to facilitate protein degradation, synaptic remodeling, synaptic plasticity, and memory consolidation. Although based largely on circumstantial evidence from the E_2_ and UPS literatures, our model provides a framework to empirically test the roles of several UPS mechanisms in the effects of E_2_ on memory in both sexes across multiple brain regions and subcellular sites.

## Discussion

This review has summarized evidence suggesting that protein degradation is an important regulator of synaptic plasticity and memory (Kaang and Choi, 2012; Hegde, 2017), yet the role that UPS-mediated protein degradation plays in regulating E_2_’s modulatory effects on memory formation in either sex remains unexplored. E_2_ may facilitate hippocampal structural plasticity and memory consolidation in part by regulating protein degradation mediated by the UPS. This hypothesis is supported by evidence that E_2_ can directly stimulate UPS activity in hippocampal slices (Briz et al., 2015), cultured neurons (Lai et al., 2019), and in a rat model of long-term E_2_ deprivation (Zhang et al., 2011). This notion is further strengthened by data suggesting that the UPS is regulated in part by sex steroid hormones, such as E_2_, as males and females appear to have different baseline regulation of, and requirement for, proteasome activity following fear learning, and target different proteins for proteasomal degradation after learning (Devulapalli et al., 2019, 2021; Farrell et al., 2021; Martin et al., 2021). As such, there is sufficiently plausible evidence to support future studies exploring a role for the UPS in estrogenic memory modulation.

When speculating why E_2_ might stimulate UPS-mediated protein degradation to regulate hippocampal memory formation, we have proposed that E_2_ triggers degradation of proteins in the synapse to promote structural remodeling of CA1 dendritic spines. However, E_2_ may also stimulate UPS activity to compensate for the enhanced synaptic potentiation caused by E_2_ administration. Interestingly, some evidence suggests that E_2_ activates the UPS to regulate the expression of proteins involved in synaptic transmission. For example, GluA1-containing AMPA receptors are ubiquitinated and degraded in the CA3 region of the male rat hippocampus following E_2_ administration (Briz et al., 2015). Similarly, the pore-forming subunit of L-type voltage gated calcium channel, Cav1.2, is ubiquitinated and degraded in cultured primary cortical neurons in an ERα-dependent manner (Lai et al., 2019). These findings might suggest that E_2_ can also regulate UPS activity in a manner that promotes homeostasis following E_2_-induced excitatory synaptic potentiation. It is of course possible that E_2_ can promote UPS activity in a manner to support both structural remodeling of synapses and to permit cellular homeostasis. However, the time course of E_2_-UPS interactions for the latter would likely occur at a time point later than 30 min, as we have proposed for structural remodeling.

Future work should examine the extent to which E_2_ requires UPS activity in males and females to support changes in hippocampal plasticity and memory. To our knowledge, no studies to date have examined whether sex differences exist in the requirement for proteasome activity in males and females following non-aversive forms of learning. As such, it remains unclear whether non-aversive tasks, such as the object placement and object recognition paradigms, activate the same cell signaling mechanisms and proteasome subunits to upregulate protein degradation in males and females that have been documented in aversive tasks. Work in this direction could potentially reveal critical baseline and learning-induced sex differences that may provide an impetus to examine the contributions of sex steroid hormones.

Moreover, we speculate that the cellular mechanisms that signal a need for protein degradation might differ between males and females, as previous work from our lab and others demonstrates that the molecular mechanisms through which E_2_ mediates DH plasticity and memory consolidation differ between the sexes (Oberlander and Woolley, 2016; Koss et al., 2018; Jain et al., 2019; Koss and Frick, 2019). Much less is known about the potential time course through which E_2_ requires UPS activity, although data suggest that the need for protein degradation during consolidation overlaps with that for protein synthesis (Park and Kaang, 2019), potentially indicating that E2 stimulates UPS activity rapidly following DH infusion, as we previously documented for local protein synthesis (Fortress et al., 2013; Tuscher et al., 2016). Finally, it remains unclear which proteins could be targeted within the DH following E_2_ treatment, and whether these protein targets differ between the sexes. Future studies in this realm would provide more direct insights into how E_2_ modifies the existing molecular framework to support hippocampal plasticity and memory.

In conclusion, this review has provided an overview of the signaling mechanisms so far identified as critical for E_2_ and UPS function, with particular emphasis on the ways in which these mechanisms overlap to support structural integrity and protein composition of hippocampal synapses. If UPS activity is integral to E_2_’s effects on memory, then this could lead to exciting new avenues of basic research into hormonal regulation of cognition that could have important clinical implications for treating psychiatric and neurodegenerative diseases in which sex or E_2_ play a role.

## Data Availability Statement

The original contributions presented in the study are included in the article, further inquiries can be directed to the corresponding author.

## Ethics Statement

The animal study was reviewed and approved by University of Wisconsin–Milwaukee Institutional Animal Care and Use Committee.

## Author Contributions

KF and SB conceived the hypothesis that is the focus of the manuscript. KF and SB were both responsible for reviewing relevant literature, preparing the manuscript, and approving the submitted version. Both authors contributed to the article and approved the submitted version.

## Conflict of Interest

KF is a co-founder of, and shareholder in, Estrigenix Therapeutics, Inc., a company which aims to improve women’s health by developing safe, clinically proven treatment for the mental and physical effects of menopause. She also serves as the company’s Chief Scientific Officer. The remaining author declares that the research was conducted in the absence of any commercial or financial relationships that could be construed as a potential conflict of interest.

## Publisher’s Note

All claims expressed in this article are solely those of the authors and do not necessarily represent those of their affiliated organizations, or those of the publisher, the editors and the reviewers. Any product that may be evaluated in this article, or claim that may be made by its manufacturer, is not guaranteed or endorsed by the publisher.

## Abbreviations

E_2_: 17β-estradiol
DH: dorsal hippocampus
BLA: basolateral amygdala
LTP: long-term potentiation
UPS: ubiquitin proteasome system
CA1: cornu ammonis 1
CA3: cornu ammonis 3
ER(s): estrogen receptor(s)
ERα: estrogen receptor alpha
ERβ: estrogen receptor beta
GPER: G-protein-coupled estrogen receptor
mGluRs: metabotropic glutamate receptors
mGluR1a: metabotropic glutamate receptor 1a
NMDA: N-methyl-D-aspartate
NMDAR(s): NMDA receptor(s)
AMPA: α-Amino-3-hydroxy-5-methyl-4-isoxazolepropionic acid
ERK: extracellular signal-regulated kinase
PI3K: phosphoinositide 3-kinase
mTOR: mechanistic target of rapamycin
mTORC1: mechanistic target of rapamycin complex 1
JNK: C-jun N-terminal kinase
PKA: protein kinase A
CaMKII: calcium/calmodulin-dependent protein kinase II
PSD: post-synaptic density
CP: core particle
RP: regulatory particle
K48: lysine 48
Rpt6: regulatory particle triple-ATPase 6 subunit
cAMP: cyclic adenosine monophosphate
LTF: long-term facilitation
CREB: cAMP-response element binding protein
ATF4: activating transcription factor 4
β-lac: clasto-lactacystin β-lactone
Lac: lactacystin
TUBE: tandem ubiquitin binding entity.
